# Patterns of multimorbidity and some psychiatric disorders: A systematic review of the literature

**DOI:** 10.3389/fpsyg.2022.940978

**Published:** 2022-09-16

**Authors:** Luis Fernando Silva Castro-de-Araujo, Fanny Cortes, Noêmia Teixeira de Siqueira Filha, Elisângela da Silva Rodrigues, Daiane Borges Machado, Jacyra Azevedo Paiva de Araujo, Glyn Lewis, Spiros Denaxas, Mauricio L. Barreto

**Affiliations:** ^1^Center of Data and Knowledge Integration for Health (CIDACS), Fiocruz, Bahia, Brazil; ^2^Department of Psychiatry, Austin Health, The University of Melbourne, Parkville, VIC, Australia; ^3^Department of Health Sciences, University of York, York, United Kingdom; ^4^Federal University of Ceará, Ceará, Brazil; ^5^Department of Global Health and Social Medicine, Harvard Medical School, Boston, MA, United States; ^6^Division of Psychiatry, University College London, London, United Kingdom; ^7^Institute of Health Informatics, University College London, London, United Kingdom

**Keywords:** multimorbidity, psychiatric disorders, clustering, aging, patterns

## Abstract

**Objective:**

The presence of two or more chronic diseases results in worse clinical outcomes than expected by a simple combination of diseases. This synergistic effect is expected to be higher when combined with some conditions, depending on the number and severity of diseases. Multimorbidity is a relatively new term, with the first fundamental definitions appearing in 2015. Studies usually define it as the presence of at least two chronic medical illnesses. However, little is known regarding the relationship between mental disorders and other non-psychiatric chronic diseases. This review aims at investigating the association between some mental disorders and non-psychiatric diseases, and their pattern of association.

**Methods:**

We performed a systematic approach to selecting papers that studied relationships between chronic conditions that included one mental disorder from 2015 to 2021. These were processed using Covidence, including quality assessment.

**Results:**

This resulted in the inclusion of 26 papers in this study. It was found that there are strong associations between depression, psychosis, and multimorbidity, but recent studies that evaluated patterns of association of diseases (usually using clustering methods) had heterogeneous results. Quality assessment of the papers generally revealed low quality among the included studies.

**Conclusions:**

There is evidence of an association between depressive disorders, anxiety disorders, and psychosis with multimorbidity. Studies that tried to examine the patterns of association between diseases did not find stable results.

**Systematic review registration:**

https://www.crd.york.ac.uk/prospero/display_record.php?ID=CRD42021216101, identifier: CRD42021216101.

## Introduction

The management of chronic diseases is one of the main challenges faced by health care systems today. Chronic conditions, when they happen together, seem to interact and worsen each other's clinical courses (Mendenhall et al., 2017). This effect is expected to be higher with some combinations of conditions more than others, and with number and severity of diseases (Mendenhall, 2017). However, most research done in the relations between chronic conditions consider them in pairs, within a hierarchy (co-morbid). This paradigm is aligned to the way the biomedical model organizes training of health providing professionals, with each medical specialist within the boundaries of their own field (World Health Organization, 2016). This introduces an arbitrary division and ignores patterns of association that may be clinically relevant and could help reduce morbidity if early addressed by practitioners (MacMahon, 2018). On the other hand, the multimorbidity (MM) concept tries to shift toward a system where there are no index conditions, and the subject of interest is rather the interaction between diseases.

This shift toward the MM concept has implications in several areas. It requires that specialists interact and communicate efficiently with staff from other background training (Sinyor et al., 2019). This type of more integrative care is costly (Valderas et al., 2009), may require reorganizing mental health and general practices (Sinyor et al., 2019) to a more holistic care (arguably simplified in places where a universal health system exists, such as in UK), and impacts research itself, as traditional association statistical methods are better suited for comparisons between two variables.

Most of the studies consider MM as the presence of two or more disorders. MacMahon (2018) declared MM a health priority, as its prevalence is increasing in many regions of the world over the past 20 years. The prevalence is also expected to grow with the increase in life expectancy (MacMahon, 2018). Patients affected by MM have more disability, tend to use more medications (van Oostrom et al., 2014), have worse quality of life (Ralph et al., 2013), more cognitive impairment (Koyanagi et al., 2018; Wang et al., 2020), and may die earlier than non-affected peers (Wei and Mukamal, 2018). Multimorbidity is also linked to increased use and costs of health services (van Oostrom et al., 2014).

The first two major documents on this subject are a summary of a roundtable meeting by The Academy of Medical Sciences (2015) and a report by World Health Organization (2016). This was followed by the Lancet series on Syndemics (Mendenhall et al., 2017), the creation of the Medical Subject Heading (MeSH) term for multimorbidity in National Library of Medicine (2018), the multimorbidity report by the American Academy of Medical Sciences the same year (MacMahon, 2018), and the Lancet Psychiatry Commission Blueprint for protecting the physical health of people with mental illness in 2019 (Firth et al., 2019). This impacts the slow transition of the literature into methods that more accurately assess the interactions between chronic diseases and development, or use of more suited methods to this end (like clustering methods). Furthermore, mental disorders in the context of multimorbidity have been less studied, as clinical conditions have become the main subjects of the field.

The complex relations of mental disorders and chronic diseases is not entirely understood. It is certainly expected to happen, as mental disorders are linked to worse adherence to clinical treatments (Patel and Chatterji, 2015), to lack of physical activity (Bueno-Antequera and Munguía-Izquierdo, 2020), and antipsychotics are linked to metabolic syndromes (Hálfdánarson et al., 2017). The hypothesized mechanisms through which mental disorders may be associated with chronic diseases include: (A) the metabolic syndrome resulting from psychiatric medication; (B) direct inflammatory process from harmful drug/alcohol/tobacco use; (C) childhood adversities as environmental factors for both non-communicable diseases and mental disorders (Patel and Chatterji, 2015); and (D) inflammatory mechanisms such as higher concentration levels of C-reactive protein, interleukin 6 and 12 and tumor necrosis factor in acute depression (Osimo et al., 2020). Additionally, individuals with mental disorders usually make poor lifestyle choices (with less exercises, unhealthy eating habits, tobacco use) and have less access to prevention policies and health care (Patel and Chatterji, 2015). So far studies have found high prevalence of MM with mental disorders among young individuals from deprived backgrounds compared to those resident of more affluent areas (Barnett et al., 2012), and higher rates of multimorbidity with mental disorders in women (Barnett et al., 2012). The majority of studies on MM are concentrated on older individuals from high income countries (MacMahon, 2018). More studies on younger individuals, from more deprived backgrounds are necessary to understand the association between socioeconomic drivers and MM with mental disorders. Previous systematic reviews found higher rates of MM and specific psychiatric disorders, like depression (Read et al., 2017) and schizophrenia (Rodrigues et al., 2021). However, evidence is still fragmented, lacks investigation of trends over time, and rarely uses methods that can estimate the effect of mechanisms.

This review aims at investigating the association between five prevalent mental disorders and non-psychiatric diseases (including the chronic infectious diseases: tuberculosis, and HIV), and the pattern of association between them. It also will organize information in a descriptive synthesis including studies that had multimorbidity as a focus from the start, avoiding those exclusively linked to co-morbidity.

## Methods

We reported this systematic review according to the PRISMA 2020 guideline (Page et al., 2021). The protocol of this review was registered at PROSPERO (registration number CRD42021216101). The search strategy included MeSH terms with the final expression: (multimorbidity[MeSH Terms]) AND (mental disorder[MeSH Terms] OR anxiety[MeSH Terms] OR depressive disorder[MeSH Terms] OR psychotic disorders[MeSH Terms] OR substance related disorders[MeSH Terms] AND (“2015/01/01”[Date - Entry]: “3000”[Date - Entry])). We searched PubMed and Scielo databases on 31/05/2021 for original articles published in the last 6 years (2015–2021) that studied multimorbidity and at least one of the mental disorders we listed above. We chose studies published from 2015 because that was the year that the Medical Science Academy held the meeting, which led to the “Multimorbidity: a priority for global health research” report in 2018. We also reviewed references from relevant prior systematic reviews, in order to identify any other eligible studies. We selected original studies published in English, Portuguese or Spanish. Furthermore, we report results in a descriptive synthesis, including a quality assessment of the studies.

### Eligibility criteria

Any quantitative article that analyzed the association between five psychiatric disorders (depression, anxiety, post-traumatic stress disorder-PTSD, substance use disorder and psychosis) and chronic physical conditions (infectious or non-communicable) were included. In addition to this, studies analyzing multimorbidity patterns that included mental disorders were also added. We excluded gray literature, studies focused on statistical models with no mention of multimorbidity patterns or association and studies that examined prevalence only. Therefore, the minimal definition of MM for this review was the presence of one chronic condition plus one of the five psychiatric disorders listed above.

### Screening and data extraction

We performed screening of title and abstract, full text review and data extraction with the Covidence web tool (Veritas Health Innovation, 2021). Two reviewers (NTSF and FC) independently screened titles and abstracts. We discussed the disagreements to reach a consensus. The full text screening followed the same approach. Reasons for exclusion were: wrong outcome (studies reporting only prevalence) or wrong study design (modeling study).

### Data extraction and risk of bias

Two reviewers (NTSF and FC) independently extracted data from included studies, with a third reviewer (JAPA) establishing comparison and consensus. We created a data extraction form including information on study characteristics, participant characteristics, setting, statistical method, outcomes, main conclusions and limitations.

We assessed the quality of each included article using the Newcastle-Ottawa Scale (NOS) (Wells et al., 2000) for observational studies. For cohort studies, we used the thresholds for converting the Newcastle-Ottawa scales to Agency for Health Research and Quality (AHRQ) standards: good, fair, and poor quality. We did not assign scores for cross-sectional studies. For these studies, we adapted the scale and included bias listed by the study authors. We did not evaluate the quality of systematic reviews and meta-analysis.

### Data analysis

We tabulated our findings as a narrative synthesis, describing the main characteristics of the studies and critical findings. Furthermore, we focused on the association between multimorbidity and psychiatric disorders by using crude and/or adjusted relative risk (RR), risk difference (RD) or odds ratio reported by the studies.

## Results

**Figure 1:**
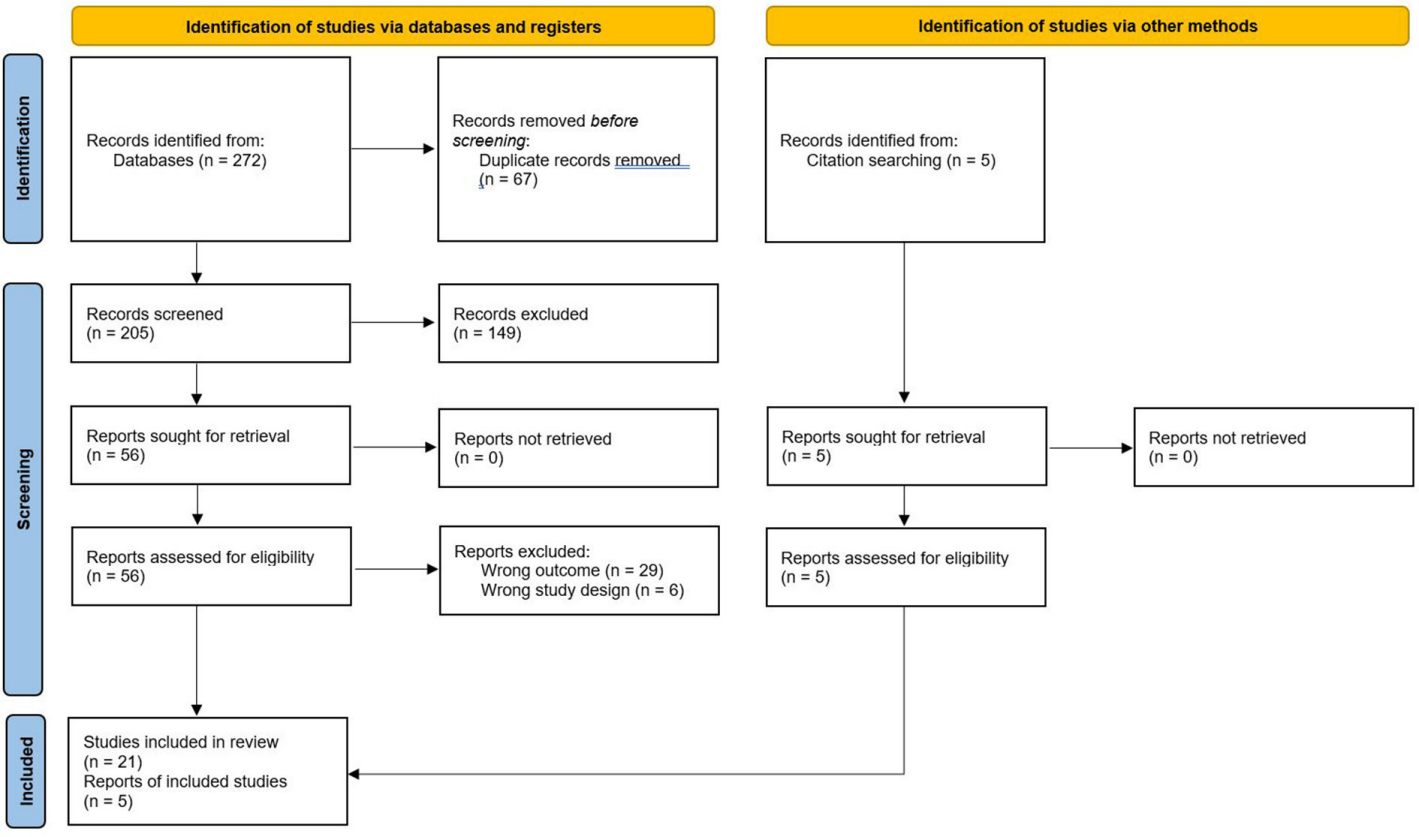
PRISMA flow diagram. *From:* Page et al. (2021).

We screened 205 titles and abstracts for eligibility. After reviewing the full text of 56 articles, we included 26 studies in our review (Figure 1). Six articles (23%) were multi-country studies; five (19%) from the USA, two (8%) from China, South Africa and Spain. One (4%) from each Bahrain, Brazil, Canada, Croatia, UK, Italy, Netherlands, Pakistan, and Switzerland. The papers included discussed the following psychiatric conditions: depression, anxiety, bipolar disorder, dysthymia, adjustment and personality disorder, psychosis, schizophrenia, post-traumatic stress disorder (PTSD), dementia, eating disorder dissociative and somatoform disorders, intellectual disabilities, impulse-control, premenstrual dysphoria, insomnia, alcohol abuse, tobacco and substance use disorders. The most common chronic conditions were cardiovascular, respiratory and renal diseases, diabetes, hepatitis and obesity (Table 1). Table 2 summarizes the main findings for the association between multimorbidity and psychiatric disorders. We provided a narrative synthesis below according to specific psychiatric disorders.

**Table 1:** Study characteristics.

**References**	**Country**	**Psychiatric disorder**	**Physical chronic** **conditions**	**Study population** **Age range**	**Sample size**	**Study design**
Li et al. (2015)	China	Depression	NR	• Adults residing in rural villages • Age range: 60–101	3,824 individuals	Observational: cross-sectional
Arokiasamy et al. (2015)	Multi-country	Depression	Angina pectoris, arthritis, asthma, chronic lung disease, diabetes mellitus, hypertension, stroke, vision impairment	• Adults aged 50 and older, with a smaller cohort of respondents aged 18–49 • Age range: 18+	42,236 individuals	Observational: cross-sectional
Stubbs et al. (2016)	Multi-country	Psychosis or subclinical psychosis	Arthritis, angina pectoris, asthma, diabetes, chronic back pain, visual impairment, hearing problems, edentulism, tuberculosis	• Adults with a valid home address • Age range: 18–65	242,952 individuals	Observational: cross-sectional
Gould et al. (2016)	USA	Anxiety and depression	Arthritis, cancer, diabetes, heart condition, high blood pressure, lung disease, stroke	• Individuals randomly selected to complete the Psychosocial Questionnaire in the Health and Retirement Study • Age range: 65+	4,219 individuals	Observational: cross-sectional
Lenzi et al. (2016)	Italy	Schizophrenia and psychosis, bipolar disorder, depression, dysthymia, anxiety, dissociative and somatoform disorders, personality disorders, substance use disorders, intellectual disabilities, other mental disorders	Myocardial infarction, congestive heart failure, peripheral vascular disease, cerebrovascular disease, hypertension, dementia, including Alzheimer's disease, chronic pulmonary disease, rheumatic disease, liver disease, diabetes, paralysis, other neurological disorders, renal disease, any malignancy, including lymphoma and leukemia, except malignant neoplasm of skin, metastatic solid tumor and AIDS/HIV.	• Adults residents of Emilia-Romagna region on 31 December 2012 • Age range: 18+	3,759,836	Observational: cross-sectional
Read et al. (2017)	Multi-country	Depression	NR	• Individuals with and without multimorbidity and with no chronic condition • Age range: 15+	40 studies	Systematic review and meta-analysis
Stubbs et al. (2017)	Multi-country	Depression	Tuberculosis, visual impairment, hearing problem, chronic back pain, edentulism, arthritis, angina, asthma and diabetes.	• Individuals with a valid home address • Age range: 18+	190,593 individuals	Observational: cross-sectional
Holvast et al. (2017)	Netherland	Depression	AIDS and HIV infection, malignancy, visual disorders, hearing disorders, congenital cardiovascular anomaly, disorders of endocard/valvular heart disease, heart failure, coronary heart disease, arrhythmias, stroke, rheumatoid arthritis, peripheral arthritis, chronic neck and back pain, osteoporosis, Parkinson's disease, epilepsy, migraine, chronic obstructive pulmonary disease, asthma, diabetes mellitus	• Patients with late-life depression in primary care • Age range: 60+	4,477 individuals	Observational: cross-sectional
DiNapoli et al. (2017)	USA	Mood Disorders: depressive and anxiety disorders	Endocrine, digestive, circulatory, respiratory	• Veterans who received primary care through the VA Pittsburgh Healthcare System (VAPHS) • Age range: 50+	34,786 individuals	Observational: cross-sectional
Gabilondo et al. (2017)	Basque Country (Spain)	Schizophrenia, dementia	Hypertension, ischemic heart disease, Parkinson, diabetes, viral hepatitis, HIV, chronic pulmonary disease, migraine, osteoarticular disorders, cardiovascular conditions, among others.	• Individuals covered by public health insurance in 31st August 2011 and who had been covered for at least 6 months in the previous year • Age range: 16+	2,255,406 individuals	Observational: cross-sectional
Jahrami et al. (2017)	Bahrain	Schizophrenia	Cardiovascular disease, type 2 diabetes, hypertension, obesity, musculoskeletal Disorder	• Cases: recruited from Psychiatric Hospital, Bahrain • Controls: recruited from primary health centers, and were free from serious mental illness • Age range: 20–60	240 individuals	Observational: case-control
Vancampfort et al. (2017)	Multi-country	Anxiety	Angina, arthritis, asthma, chronic back pain, diabetes, edentulism, hearing problem, tuberculosis, and visual impairment	• Community-dwelling adults • Age range: 18+	181,845 individuals	Observational: cross-sectional
Jacob et al. (2018)	UK	Post-traumatic stress disorder, alcohol dependence, drug use, disordered eating, anxiety and depression as mediators	Cancer, diabetes, epilepsy, migraine, cataracts/eyesight problems, ear/hearing problems, stroke, heart attack/angina, high blood pressure, bronchitis/emphysema, asthma, allergies, stomach ulcer or other digestive problems, liver problems, bowel/colon problems, bladder problems/incontinence, arthritis,	• English adult population, residing in private households • Age range: 18+	7,403 individuals	Observational: cross-sectional
			bone/back/joint/muscle problems, infectious disease, and skin problems			
Filipčić et al. (2018)	Croatia	Any psychiatric disorder	Hypertension, urinary incontinence, obesity, spine and back pain, neck spine, allergies, asthma, diabetes, chronic obstructive pulmonary disease, kidney problems, cirrhosis of the liver, myocardial infarction, arthrosis, stroke, coronary heart disease	• Patients diagnosed with any psychiatric disorder who were treated in a psychiatric hospital as in-patients or outpatients and have permanent residency in the city of Zagreb or Zagreb County. General population of Croatian citizens living in private households in the city of Zagreb and Zagreb County • Age range: 18+	1,897 individuals	Observational: cross-sectional
Peltzer (2018)	South Africa	Tobacco and Alcohol-use disorder, anxiety or depressive disorders, symptoms of post-traumatic stress disorder	Myocardial infarction or angina pectoris, arthritis, asthma, chronic lung disease, type 2 diabetes, hypertension, dyslipidaemia, malignant neoplasms, tobacco and alcohol-use disorder	• New and retreatment tuberculosis patients within 1 month of anti-TB treatment being initiated from high TB caseloads primary health care facilities • Age range: 18+	4,207 individuals	Observational: cross-sectional
MacLean et al. (2018)	USA	Alcohol use disorder and tobacco use disorder, dementia, schizophrenia, bipolar disorder, major depression, other depression, post-traumatic stress disorder, anxiety disorder, adjustment disorder, personality disorder, and other psychiatric diagnosis, substance use disorder	Hepatitis and renal disease	• All veterans who had received a diagnosis of either alcohol or tobacco use disorders or both during 2012 • Age range: not informed	988,674 individuals	Observational: Cohort
Violan et al. (2018)	Spain	Mental and behavioral disorders due to psychoactive substance use, stress-related and somatoform disorders	Metabolic disorders and other physical conditions: non-inflammatory disorders of female genital tract, hypertensive disease, other soft tissue disorders, disorders of thyroid gland, benign neoplasms	• Patients with multimorbidity attended in primary care centers • Age range: 45–64	408,994	Observational: cross-sectional
Farooq et al. (2019)	Pakistan	Anxiety and depressive symptoms	Hypertension, obesity, dyslipidaemia, diabetes, heart diseases, stroke, migraines, asthma and chronic obstructive pulmonary disease, anemia, thyroid disease, diseases of bones and joints, dyspepsia/peptic ulcer, hepatitis B or C, chronic kidney diseases including stones, cancer, disability	• Adults living in the Gulshan-e-Iqbal town of Karachi, Pakistan • Age range: 30+	3,250 individuals	Observational: cross-sectional
Han et al. (2019)	USA	Substance use: cannabis, cocaine, methamphetamine, heroin, inhalants, hallucinogens, and prescription drug misuse and substance use disorder	Asthma, bronchitis, chronic obstructive pulmonary disease, cirrhosis, diabetes, heart conditions, hepatitis, high blood pressure, cancer, kidney disease, and HIV/AIDS	• Non-institutionalized individuals with substance use disorder (SUD) and past-year use • Age range: 18+	85,701 individuals	Observational: cross-sectional
Crawford and Thornton (2019)	USA	Alcohol use, depression, anxiety disorder, and bipolar disorder	HIV	• People living with HIV patients of an urban infectious disease clinic • Age range: not informed	1,635 individuals	Observational: cohort
Romain et al. (2019)	Canada	Major depressive episode, mood disorders and anxiety disorders	Obesity	• People with obesity • Age range: 18+	1,315 individuals	Observational: cross-sectional
Petersen et al. (2019)	South Africa	Depression and alcohol use disorders	Hypertension, diabetes	• Patients attending three large health facilities in the Dr Kenneth Kaunda district • Age range: 18+	2,549 individuals	Observational: cross-sectional
Wang et al. (2019)	Brazil	Anxiety, mood, impulse-control, and substance use disorders, plus premenstrual dysphoria (in women) and heavy drinking.	Cardiovascular diseases, hypertension, diabetes mellitus, arthritis, chronic musculoskeletal pain, headache or migraine, digestive, respiratory, neurological diseases, and cancer.	• Urban sample of non-elderly adults • Age range: 18–64	2,713	Observational: cross-sectional
Aubert et al. (2019)	Switzerland	Mood disorders, substance-related disorders, anxiety disorders	Chronic heart disease, Cerebrovascular disease, Hematological malignancy, chronic kidney disease, chronic obstructive pulmonary disease and bronchiectasis, pulmonary heart disease, peripheral and visceral atherosclerosis, paralysis, arthropathy and arthritis,	• Adults discharged from general hospital • Age range: not informed	42,739	Observational: cohort
			cerebrovascular disease, acute and unspecified renal failure, solid malignancy, other nervous system disorders, other and ill-defined heart disease, thyroid disorders, nephritis, nephrosis and renal sclerosis, diseases of white blood cells			
Wong et al. (2020)	China	Depression, anxiety, insomnia	NR	• Older adults who had ≥ 2 chronic conditions, recruited from four public primary care clinics. • Age range: 60+	583 individuals	Observational: cohort
Rodrigues et al. (2021)	Multi-country	Psychotic disorders	NR	• Individuals with psychotic disorders • Age range: not informed	14 studies	Systematic review and meta-analysis

**Table 2:** Study results.

**References**	**Main outcome**	**Statistical method**	**Results**
Li et al. (2015)	Association between depressive symptoms and chronic conditions and socioeconomic status	Multilevel logistic regression, odds ratio	Unadjusted model Association depressive symptoms and chronic conditions OR = 1.379 (95% CI: 1.291 to 1.473), *p* < 0.001
Arokiasamy et al. (2015)	Association between multimorbidity and four primary health outcomes: lower self-rated health, depression, limitation in activities of daily living, and poorer quality of life	Multilevel multinomial logit model, odds ratio, beta coefficient	Disease count on depression Unadjusted model 1 disease: OR = 1.77 (95% CI: 1.57 to 2.01) 2 diseases: OR = 2.80 (95% CI: 2.48 to 3.18) 3 diseases: OR = 4.74 (95% CI: 4.12 to 5.46) 4+ diseases: OR = 8.75 (95% CI: 7.53 to 10.12) All *p <* 0.01 Adjusted model 1 disease: OR 1.62 (95% CI: 1.42 to 1.84) 2 diseases: OR 2.44 (95% CI: 2.14 to 2.82) 3 diseases: OR 4.05 (95% CI: 3.47 to 4.75) 4+ diseases: OR 7.33 (95% CI: 6.24 to 8.61) All *p* < 0.01
Stubbs et al. (2016)	Association between psychosis and multimorbidity	Multivariable logistic regression, odds ratio	Adjusted model Subclinical psychosis: OR = 2.20 (95% CI: 2.02 to 2.39) Psychosis diagnosis: OR = 4.05 (95% CI: 3.25 to 5.04) All *p* < 0.001
Gould et al. (2016)	Association between the number of medical conditions and elevated anxiety or depression	Multiple logistic regression analysis, odds ratio	Unadjusted model 1 condition and anxiety: OR = 1.24 (95% CI: 0.70 to 2.21), non-significant 2 conditions and anxiety: OR = 1.96 (95% CI: 1.13 to 3.41), *p* < 0.001 3 or more conditions and anxiety: OR = 3.49 (95% CI: 2.05 to 5.95), *p* < 0.001 Wald F (3,4181) = 22.46 Adjusted model 1 condition and anxiety: OR = 1.21 (95% CI: 0.67 to 2.16), non-significant 2 conditions and anxiety: OR = 1.79 (95% CI: 1.02 to 3.12), *p* < 0.001 3 or more conditions and anxiety: OR = 3.04 (95% CI: 1.77 to 5.21), *p* < 0.001 Wald F (3,4181) = 17.08
Lenzi et al. (2016)	Descriptive analysis of differences in the prevalence of multimorbidity in relation to age, gender, and citizenship	Exploratory factor analysis, tetrachoric correlation matrix for factor analysis	Multimorbidity pattern—(1) Psychiatric disorders: Schizophrenia and psychosis 0.60, Anxiety, dissociative, and somatoform disorders 0.54, Depression 0.44; (4) Liver diseases, AIDS/HIV and substance use: Substance use disorders 0.55
Read et al. (2017)	Risk of depressive disorder in people with and without multimorbidity	Meta-analysis risk and odds ratios	Risk for depressive disorder for people with multimorbidity compared to those without multimorbidity RR = 2.13 (95% CI: 1.62 to 2.80), *p* < 0.001 Risk of having depressive disorder in people with multimorbidity compared to those with no chronic physical condition RR = 2.97 (95% CI: 2.06 to 4.27), *p* < 0.001 Odds of having a depressive disorder with increasing number of chronic physical conditions OR = 1.45 (95% CI: 1.28 to 1.64), *p* < 0.001 Correlation between number of chronic physical conditions and depressive symptoms: r = 0.26 (95% CI: 0.18–0.33), *p* < 0.001
Stubbs et al. (2017)	Association between the whole depressive spectrum and multimorbidity	Multivariable logistic regression, odds ratio	Unadjusted model Subsyndromal depression: OR =2.62 (95% CI: 2.17 to 3.15) Brief depressive episode: OR = 2.14 (95% CI: 1.84 to 2.48) Depressive episode: OR = 3.44 (95% CI: 3.12 to 3.79) All *p* < 0.0001 Adjusted model Overall (43 countries): OR = 3.26 (95% CI: 2.98 to 3.57); I-squared=58.1%, p=0.000
Holvast et al. (2017)	Associations between patients diagnosed with late-life depression in primary care and multimorbidity and polypharmacy	Multivariable, multilevel, negative binomial regression, prevalence ratio and odds ratio	Adjusted model Association depression and multimorbidity: OR = 1.55 (95% CI: 1.33 to 1.81), *p* < 0.001 Association psychological diagnoses and multimorbidity : OR = 1.34 (95% CI: 1.15 to 1.57), *p* < 0.001
DiNapoli et al. (2017)	Prevalence of and relationship between mood disorders and multimorbidity in middle-aged and older veterans	Binary logistic regression, odds ratio chi-square, Wald chi-square	Unadjusted model Predictors of Having a Depressive and/or Anxiety Disorder, organ system with chronic disease count (reference: 0–2) 3–5 chronic diseases: OR = 1.70 (95%CI: 1.21 to 2.40), *p* = 0.002 6–7 chronic diseases: OR = 2.56 (95%CI: 1.83 to 3.59), *p* < 0.001 8–9 chronic diseases: OR = 4.05 (95%CI: 2.89 to 5.66), *p* < 0.001 10–13 chronic diseases: OR = 6.62 (95%CI: 4.73 to 9.25), *p* < 0.001
Gabilondo et al. (2017)	Epidemiology of comorbidities with chronic physical conditions in schizophrenia	Cluster analysis, chi-square test	Unadjusted model Comorbidities with physical conditions, schizophrenia vs. controls 1 chronic physical illness: OR = 1.64 (95% CI: 1.56 to 1.73) 2 chronic physical illnesses: OR = 1.63 (95% CI: 1.53 to 1.75) 3 or more chronic physical illnesses: OR = 1.12 (95% CI: 1.05 to 1.19) All *p* < 0.001 Specific diseases OR (95% CI) Parkinson: 47.89 (44.49–51.55) Dementia: 4.86 (4.37–5.40) Viral hepatitis: 3.31 (2.87–3.82) HIV: 2.25 (1.66–3.05) Diabetes: 2.23 (2.06–2.40) Chronic pulmonary disease: 2.10 (1.88–2.35) Chronic liver or pancreatic disease: 2.06 (1.79–2.36) Psoriasis or eczema: 1.81 (1.35–2.42) Hypothyroidism: 1.72 (1.57–1.88) Dyspepsia: 1.55 (1.41–1.70) Epilepsy (currently treated): 1.44 (1.11–1.88) Heart failure: 1.37 (1.09–1.73) Blindness & low vision: 1.20 (1.02–1.42) Asthma (currently treated): 0.84 (0.72–0.98) Hypertension: 0.78 (0.73–0.84) Ischemic heart disease: 0.78 (0.65–0.94) Cancer: 0.76 (0.65–0.88) Prostatic hypertrophy: 0.75 (0.61–0.91)
			Peripheral neuropathy: 0.72 (0.62–0.83) Diverticular disease of intestine: 0.66 (0.51–0.87) Gout: 0.66 (0.53–0.84) Atrial fibrillation: 0.65 (0.52–0.81) Degenerative joint disease: 0.63 (0.55–0.73) Rheumatoid arthritis and autoimmune and connective tissue diseases: 0.53 (0.40–0.70) Osteoporosis: 0.51 (0.42–0.63) Migraine: 0.36 (0.20–0.64) All *p* < 0.001
Jahrami et al. (2017)	Impact of the presence of one or more dietary and lifestyle risk factors on the comorbidities among patients with schizophrenia in comparison to controls	Logistic regression, odds ratio	Schizophrenia vs. controls 1 comorbidity: OR = 3.7 (95%CI: 2.0 to 6.9) 2 comorbidities: OR = 3.3 (95%CI: 1.8 to 6.0) ≥3 comorbidities: OR = 3.2 (95%CI: 1.4 to 7.7) All statistically significant Specific diseases Type 2 diabetes mellitus: OR = 4.7 (95% CI: 1.8 to 13.0) hypertension: OR = 3.1 (95% CI: 1.4 to 7.2) MSD: OR = 2.0 (95% CI: 1.1 to 4.0) Obesity: OR = 1.7 (95% CI: 0.9 to 3.3) All statistically significant
Vancampfort et al. (2017)	Associations of each chronic physical condition and number of chronic physical conditions with anxiety	Multivariable logistic regression, odds ratio	Associations between chronic physical conditions and anxiety Model 1: adjusted for sex, age, wealth, and country 1 physical condition: OR = 2.08 (95% CI: 1.90 to 2.27) 2 physical conditions: OR = 3.10 (95% CI: 2.79 to 3.45) 3 physical conditions: OR = 4.54 (95% CI: 3.90 to 5.30) 4 physical conditions: OR = 6.79 (95% CI: 95.44 to 8.47) 5+ physical conditions: OR = 9.66 (95% CI: 6.88 to 13.57)
			All *p* < 0.001 Model 2: adjusted for sex, age, wealth, depression, and country 1 physical condition: OR = 1.94 (95% CI: 1.76 to 2.13) 2 chronic physical conditions: OR = 2.63 (95% CI: 2.34 to 2.96) 3 chronic physical conditions: OR = 3.56 (95% CI: 3.00 to 4.22) 4 chronic physical conditions: OR = 4.69 (95% CI: 3.64 to 6.04) 5+ chronic physical conditions: OR = 5.49 (95% CI: 3.73 to 8.09) All *p* < 0.001 Specific diseases Model 1 Angina: OR = 2.35 (95% CI: 2.16 to 2.54) Arthritis: OR = 1.74 (95% CI: 1.59 to 1.90) Asthma: OR = 1.78 (95% CI: 1.57 to 2.02) Chronic back pain: OR = 2.67 (95% CI: 2.41 to 2.97) Diabetes: OR = 1.99 (95% CI: 1.69 to 2.34) Edentulism: OR = 1.14 (95% CI: 1.01 to 1.29) Hearing problems: OR = 1.63 (95% CI: 1.42 to 1.87) Tuberculosis: OR = 2.29 (95% CI: 1.84 to 2.84) Visual impairment: OR = 4.12 (95% CI: 3.36 to 5.04) All *p* < 0.001 Model 2 Angina: OR = 1.97 (95% CI: 1.79 to 2.16) Arthritis: OR = 1.54 (95% CI: 1.39 to 1.70) Asthma: OR = 1.56 (95% CI: 1.36 to 1.79) Chronic back pain: OR = 2.23 (95% CI: 1.99 to 2.50) Diabetes: OR = 1.83 (95% CI: 1.50 to 2.22) Edentulism: OR = 1.13 (95% CI: 0.97 to 1.31) Hearing problems: OR = 1.50 (95% CI: 1.29 to 1.74) Tuberculosis: OR = 1.85 (95% CI: 1.29 to 1.74) Visual impairment: OR = 3.96 (95% CI: 2.95 to 4.62) All *p* < 0.001
Jacob et al. (2018)	Association between post-traumatic stress disorder and physical multimorbidity	Multivariable logistic regression, mediation analyses, odds ratio	Associations of PTSD and physical multimorbidity OR = 2.47 (95% CI: 1.71 to 3.56), *p* < 0.001 Without individuals taking antipsychotics or antidepressants OR = 2.72 (95% CI: 1.80 to 4.10), *p* < 0.001
Filipčić et al. (2018)	Differences in the prevalence and patterns of chronic physical illness and multimorbidity in the general and psychiatric population	Latent class analysis, difference in prevalence between groups, Mann-Whitney U test, I-square test, absolute risk increase and relative risk increase	Any chronic physical illness (CPI) ARI: 15% RRI: 25% All *p* < 0.001 Number of CPI (≥2) ARI: 11%; RRI: 28 Specific CPI, ARRI, RRI, *p*-value Hypertension: 11%, 41%, <0.001 Urinary incontinence: 8%, 102%, <0.001 Obesity: 7%, 36%, 0.01 Spinal and back pain: 7%, 19%, 0.003 Neck spine: 6%, 23%, 0.005 Allergies: 5%, 27%, 0.004 Asthma: 4%, 129%, <0.001 Diabetes: 4%, 48%, 0.007 COPD: 3%, 43%, 0.04 Kidney problems: 2%, 33%, 0.1 Cirrhosis of the liver: 1%, 78%, 0.17 Myocardial infection: 0%, −16%, 0.51 Arthrosis: −1%, −12%, 0.38 Stroke or its chronic consequences: −1%, −28%, 0.18 Coronary heart disease or angina pectoris: −3%, −50%, 0.001
Peltzer (2018)	Prevalence of non-communicable disease multimorbidity, its pattern and impact on adverse health outcomes among patients with tuberculosis in public primary care	Multinomial logistic regression, odds ratio	Unadjusted model Multimorbidity and psychological distress 1 NCDs: OR = 1.02 (95% CI: 0.87 to 1.19), non-significant 2 NCDs: OR = 1.29 (95% CI: 1.06 to 1.55); *p* < 0.01 3 or more NCDs: OR = 1.51 (95% CI:1.16 to 1.98); *p* < 0.01 Multimorbidity and Post-Traumatic Stress Disorder 1 NCDs: OR = 1.26 (95% CI: 1.07 to 1.48); *p* < 0.01 2 NCDs: OR = 1.58 (95% CI: 1.30 to 1.92); *p* < 0.01 3 or more NCDs: OR = 1.40 (95% CI: 1.05 to 1.86); *p* < 0.05 Adjusted model Multimorbidity and psychological distress 1 NCDs: OR = 0.95 (0.80 to 1.13) 2 NCDs: OR = 1.22 (0.99 to 1.50) 3 or more NCDs: OR = 1.34 (0.99 to 1.79) All non-significant Multimorbidity and Post-Traumatic Stress Disorder 1 NCDs: OR = 1.42 (1.18 to 1.69) 2 NCDs: OR = 1.79 (1.44 to 2.22) 3 or more NCDs: OR = 1.77 (1.30 to 2.41) All *p* <0.001
MacLean et al. (2018)	Association between Alcohol Use disorder and Tobacco Use Disorder with hepatic disease	Multivariate multinomial logistic regression, odds ratio	Unadjusted model AUD+TUD vs. TUD Hepatic disease: 2.18 (2.13 to 2.2) Diabetes mellitus: 0.81 (0.80 to 0.83) Renal disease: 0.71 (0.68 to 0.74) All *p* < 0.0001 AUD+TUD vs. AUD Hepatic disease: 1.14 (1.11 to 1.17) Diabetes mellitus: 0.60 (0.59 to 0.61) Renal disease: 0.76 (0.73 to 0.79) All *p* < 0.0001
Violan et al. (2018)	Multimorbidity patterns using a non-hierarchical cluster analysis	K-means cluster analysis	Cluster 1 (40% pop): centrality 0.8; median number of diagnoses 3; Patterns of multimorbidity similar for males and female: Metabolic disorders, Hypertensive diseases, Mental and behavioral disorders due to psychoactive substance use, other dorsopathies and Other soft tissue disorders.
Farooq et al. (2019)	Prevalence of and association between anxiety and depressive symptoms with multimorbidity	Univariate and multivariate binary logistic regressions and crude and adjusted odds ratio	Unadjusted model Presence of multimorbidity associated with anxiety and depressive symptoms COR (95% CI): 1.39 (1.18 to 1.64) Anxiety and depressive symptoms with number of chronic diseases. COR (95% CI) 1 chronic diseases: 0.95 (0.74 to 1.21) 2 chronic diseases: 1.05 (0.81 to 1.34) 3 chronic diseases: 1.30 (0.99 to 1.71) 4 chronic diseases: 2.24 (1.58 to 3.17) 5+ chronic diseases: 3.06 (1.99 to 4.7) Adjusted model Presence of multimorbidity associated with anxiety and depressive symptoms AOR (95% CI): 1.33 (1.11 to 1.58) Anxiety and depressive symptoms with number of chronic diseases AOR for all variables with *p*-value <0.250 in univariate analysis. AOR (95% CI) 1 chronic diseases: 0.98 (0.69 to 1.14) 2 chronic diseases: 0.98 (0.76 to 1.27) 3 chronic diseases: 1.20 (0.90 to 1.59) 4 chronic diseases: 1.92 (1.33 to 2.78) 5+ chronic diseases: 2.62 (1.66 to 4.13)
Han et al. (2019)	Correlates of past-year substance use among adults with chronic conditions	Bivariable and multivariable logistic regression, odds ratio	Unadjusted model Correlates of past-year substance use (reference 2 conditions) OR (95%CI), *p*-value 3–4 conditions: 0.97 (0.82, 1.15), 0.70 > 5 conditions: 0.57 (0.36, 0.91), 0.02 Adjusted model OR (95%CI), *p*-value 3–4 conditions: 1.16 (0.97, 1.39), 0.11 > 5 conditions: 0.67 (0.39, 1.15), 0.15
Crawford and Thornton (2019)	Presence of multiple comorbid conditions after an HIV diagnosis	Modified Poisson regression Crude and adjusted incidence rate ratios	Unadjusted model Alcohol use among HIV patients Former vs. Never IRR (95% CI): 2.17 (1.45 to 3.24) Current vs. Never IRR (95% CI): 1.95 (1.56 to 2.43) Drug use (yes vs. no) IRR (95% CI): 1.22 (1.01 to 1.47) Adjusted model Alcohol use: Former vs. Never aIRR (95% CI): 1.49 (0.99 to 2.24) Current vs. Never aIRR (95% CI): 1.70 (1.35 to 2.14), *p* < 0.05 Drug use (yes vs. no) aIRR (95% CI): 1.11 (0.90 to 1.38)
Romain et al. (2019)	Association between physical multimorbidity and the severity of obesity with mental health and with mental disorders	Logistic regressions, odds ratio	Adjusted model when obesity classes and physical multimorbidity were considered, the latter was preferentially associated with Poor perceived mental health: OR = 3.58 (95% CI 2.07 to 6.22) Psychological distress: OR = 3.71 (95% CI 2.14 to 6.42) Major depressive episode: OR = 5.16 (95% CI 2.92 to 9.13) Mood disorders: OR = 2.31 (95% CI 1.41 to 3.78) Anxiety disorders OR = 2.46 (95% CI 1.46 to 4.16)
Petersen et al. (2019)	Association between depression and Alcohol Use Disorder (AUD) in chronic care patients	Logistic regression, odds ratio	Unadjusted model Association between HIV and depression OR = 2.08 (95% CI: 1.53 to 2.83) Association between HIV and AUD OR = 2.19 (95% CI: 1.38 to 3.48) Association between hypertension, diabetes and depression OR = 0.45 (95% CI: 0.23 to 0.89)
Wang et al. (2019)	Patterns of physical-Mental Multimorbidity	Intraclass correlation coefficient	Pattern matrix of 12-month multimorbidity of psychiatric disorders and general medical conditions Women **Factor 1 Irritable mood and headache** Premenstrual dysphoria 0.66 Mood disorder 0.64 Anxiety disorder 0.61 Impulse-control disorder 0.47 Headache/migraine 0.43 **Factor 2 Chronic diseases and chronic pain** Hypertension −0.72 Cardiovascular disease −0.60 Diabetes mellitus −0.59 Arthritis −0.54 Musculoskeletal pain −0.37 **Factor 3 Substance use disorders** Heavy drinking 0.76 Substance use disorder 0.69 Men **Factor 2 Psychiatric disorders** Mood disorder 0.57 Anxiety disorder 0.47 Impulse-control disorder 0.66 Heavy drinking 0.39 Substance use disorder 0.61
			**Factor 3 Chronic diseases** Hypertension 0.69 Cardiovascular disease 0.55 Diabetes mellitus 0.68
Aubert et al. (2019)	Identify and quantify the most prevalent combinations of chronic co-morbidities in multimorbid patients hospitalized in internal medicine wards	Latent class analysis	Probability of patient in Group 5—psychiatric (23%) to have the multimorbidity: Cerebrovascular diseases: 1% Paralysis: 2% **Chronic heart disease: 19% Other nervous system disorders: 12%** Epilepsy, convulsions: 7% Chronic kidney disease: <1% Solid malignancy: 9% Hematological malignancy: <1% Diseases of white blood cells: <1% Nephritis, nephrosis and renal sclerosis: <1% Peripheral and visceral atherosclerosis: 3% **Arthropathy and arthritis: 12%** **COPD and bronchiectasis: 15%** Pulmonary heart disease: 4% **Substance-related disorders: 30%** **Mood disorders: 19%** **Anxiety disorders: 10%**
Wong et al. (2020)	Association between independent variables (sociodemographic data and number of chronic conditions) and dependent outcome	Univariable analysis, multiple linear regression, generalized estimating equation, odds ratio	Adjustment of pre-COVID-19 scores Multiple regression Generalized Anxiety Disorder and chronic conditions >4 chronic conditions: Î^2^ = 0.33 (95% CI: 0.32 to 0.98); not significant
Rodrigues et al. (2021)	Risk of multimorbidity among people with and without psychotic disorder	Meta-analysis, risk ratio	RR = 1.69; 95% CI: 1.37 to 2.08; I-squared = 99.7%

### Overall results

Most articles discussing multimorbidity and mental disorders were cross-sectional studies (20.76%). Two studies included were systematic reviews and meta-analysis (Read et al., 2017; Rodrigues et al., 2021). Most of the papers were multi-country studies (23%). The single country with more studies was the USA (19%). The study with the largest sample had 3,759,836 individuals (Lenzi et al., 2016) and the smaller had 240 individuals (Jahrami et al., 2017). Fifteen percent of the studies included exclusively older adults (50 years and above). Depression was the psychiatric disorder most evaluated in the reviewed studies (42%), followed by anxiety (23%), and substance abuse (19%). Only four studies addressed multimorbidity among individuals with psychosis. Samples were very heterogeneous and comprised specific groups (veterans, people living with HIV, general population of a region, patients of a psychiatric hospital, patients of primary care units, patients of tuberculosis clinic, etc.). Most study samples came from high income countries (European countries: Italy, Netherlands, Spain, Croatia and Switzerland).

### Patterns of multimorbidity

The shift from co-morbidity to the multimorbidity framework resulted in more papers being published aiming at detecting patterns of association between conditions. In particular, it seems that studies that grouped diseases together were the most common. There are a number of these methods and the ones recovered in our systematic review included factor analysis and latent class analysis (Lenzi et al., 2016; Filipčić et al., 2018; Aubert et al., 2019), but there also were papers using non-supervised clustering methods (Violan et al., 2018). In this section, we present the clusters or groupings found through these methods. Only Lenzi et al. (2016) presented *ad hoc* tests between groups and age, and Filipčić et al. (2018) performed tests between classes and an outcome. Peltzer (2018) identified three patterns of multimorbidity through a factor analysis where the third factor (eigenvalue = 1.10), named substance use disorders, had high loading on daily or almost daily tobacco use (0.81) and alcohol-use disorders (0.81). Aubert et al. (2019) identified five groups as patterns of multimorbidity, Group 5 (“psychiatric diseases”) included psychiatric and neurological disorders, along with chronic heart disease, COPD and bronchiectasis, and arthropathy and arthritis, corresponding to 23% of patients.

Wang et al. (2019) reported patterns of multimorbidity using principal component analysis (PCA) as a clustering method. They used data from a local survey in a large city of Brazil, including 2,713 subjects. The clusters of chronic conditions were distinct among women and men. Among women, the first component was labeled “irritable mood and headache” and encompassed premenstrual dysphoric, mood, anxiety, impulse-control disorders, and headache/migraine. The second component was “chronic diseases and pain” and included hypertension, cardiovascular illnesses, arthritis, diabetes, and musculoskeletal pain. The third component, “substance use,” included heavy drinking and substance use disorders (SUD). For men, the first component was labeled “chronic pain and respiratory disease,” and included headache/migraine, musculoskeletal pain, arthritis, respiratory, and digestive illnesses. The second component was named “psychiatric disorders” and included impulse-control, mood, anxiety disorders, SUD, and heavy drinking. Finally, the third component described a dimension of “chronic diseases” that contained hypertension, cardiovascular illnesses, and diabetes. Of note is their finding that age was not significantly related to the “irritable mood and headache cluster” in women and to the “chronic pain and respiratory disease” cluster in men, whereas age was significantly associated to all other clusters. Furthermore, they report area level (complementing the individual level findings just described) effect sizes and significance for some variables, notably the area violence was significantly associated with the “chronic diseases” cluster in women and with the “chronic pain” cluster in men.

Violan et al. (2018) also defined patterns of multimorbidity by sex using Multiple Correspondence Analysis (MCA) and K-means clustering. In the first cluster, in women had an exclusivity value (fraction of patients with the disease included in the cluster over the total strata of patients with the disease) of 46.1% and included mental and behavioral disorders due to psychoactive substance use (tobacco); in men, the first cluster had an exclusivity value of 35.3% and included metabolic disorders. It also found that the main cluster, for men and women, included the following chronic diseases: metabolic disorders, hypertensive diseases, mental and behavioral disorders due to psychoactive substance use, other dorsopathies and other soft tissue disorders.

Lenzi et al. (2016) using exploratory factor analysis identified 5 patterns of multimorbidity: (1) psychiatric disorders, (2) cardiovascular, renal, pulmonary and cerebrovascular diseases, (3) neurological diseases, (4) liver diseases, AIDS/HIV and substance abuse and (5) tumors. As for the association between factor scores and demographic characteristics, we found that the only correlation coefficient close to 0.30 was that between multimorbidity pattern 2 and age (Spearman's ρ = 0.27, *p* < 0.001), indicating that the presence of cardiovascular, renal, pulmonary and/or cerebrovascular diseases was more common among older than younger age groups (Spearman's ρ = 0.27, *p* < 0.001), indicating that the presence of cardiovascular, renal, pulmonary and/or cerebrovascular diseases was more common among older than younger age groups.

Filipčić et al. (2018) report results from self-reports of presence of chronic physical conditions. The authors performed a latent class analysis (LCA) to identify grouping patterns in their data and found that psychiatric patients had 27% higher age-standardized relative risk for chronic physical illness. The LCA identified four groups, labeled as follows: “Relatively healthy,” “Musculoskeletal,” “Hypertension and obesity,” and “Complex multimorbidity.” But the authors report no significant differences in multimorbidity patterns.

These findings will be discussed in more length below. However, it was remarkable how varied results were regarding the clusters recovered. For example, arthritis and other painful conditions have been reported to co-occur with depressive disorders (Miguel et al., 2012), but the algorithms used only grouped arthritis with mental disorders in one (Wang et al., 2019) of the studies. Similarly, only one study grouped mental disorders with metabolic syndrome (Violan et al., 2018), which is one association expected to be found widely due to mechanisms of metabolic alterations via psychiatric medication use. It is important to notice that the list of diseases that were actually included in any of the above papers differed between them, this further hinder comparisons between the clusters reported in these works.

### Depression

Seven studies evaluated MM in patients with depression. The studies covered the risk of depression in patients with chronic diseases in general (Li et al., 2015; Holvast et al., 2017); the association between depression and HIV, hypertension and diabetes (Petersen et al., 2019) or obesity (Romain et al., 2019) the risk of depression according to the number of chronic diseases (Arokiasamy et al., 2015) the risk of multimorbidity according to the severity of depression (Stubbs et al., 2017) and the risk of depression in patients with and without multimorbidity (Read et al., 2017).

A systematic review and meta-analysis found that individuals with multimorbidity had more than twice the risk (RR 2.13 95% CI: 1.62 to 2.80; *p* < 0.001) of having a depressive disorder compared to individuals without multimorbidity (Read et al., 2017). In our review, we found two studies investigating the association between depressive symptoms and chronic diseases among people 60 years or older. In China, a cross-sectional study showed significant association (OR = 1.379 [95% CI: 1.291 to 1.473]) (Li et al., 2014) and in the Netherlands, they found a significant association in a primary care sample (OR = 1.55 [95% CI: 1.33 to 1.81]) (Holvast et al., 2017).

We also found one study investigating the association of depression with a chronic infectious disease. In South Africa, HIV was associated with depression (OR = 2.08 [95% CI: 1.53 to 2.83]) while patients with hypertension and diabetes were less likely to have depression (OR = 0.45 [95% CI: 0.23 to 0.89]) (Petersen et al., 2019). In Canada, individuals with physical multimorbidity had higher odds of major depressive episode (OR = 5.16 [95% CI 2.92 to 9.13]) (Romain et al., 2019). Arokiasamy et al. (2015) performed a multi-country study in the adult population (most aged 50 years or older) showing that the odds of depression increased significantly according to the number of chronic diseases (adjusted OR increased from 1.62 [95% CI: 1.42 to 1.84] for one disease to 7.33 [95% CI: 6.24 to 8.61] for four or more diseases) (Arokiasamy et al., 2015). Another multi-country study found that the severity of depression influenced the odds of multimorbidity. Compared with patients with no depression, the odds of multimorbidity was 2.62 times higher (95% CI: 2.17 to 3.15; *p* < 0.0001) for patients with subsyndromal depression and 3.44 times higher (95% CI: 3.12 to 3.79; *p* < 0.0001) for patients with depressive episode (Stubbs et al., 2017).

### Anxiety

Four studies addressed anxiety and multimorbidity with three of them evaluating the risk of anxiety according to the number of chronic diseases (Vancampfort et al., 2017; Romain et al., 2019; Wong et al., 2020). A fourth study assessed the risk of anxiety in individuals with obesity. In the USA, anxiety was associated with an increased number of medical diseases in adults aged 65 or older (two medical conditions, adjusted OR = 1.79 [95% CI: 1.02 to 3.12]; three or more medical diseases, adjusted OR = 3.04 [95% CI: 1.77 to 5.21]; all *p* < 0.001) (Gould et al., 2016). Vancampfort et al. (2017) performed a multi-country cross-sectional study with findings indicating that individuals with five or more physical diseases had higher odds of anxiety (adjusted OR 9.66 [95% CI: 6.88 to 13.57]; *p* < 0.001) compared to those with one physical condition (adjusted OR 2.08 [95% CI: 1.90 to 2.27]; *p* < 0.001) (Vancampfort et al., 2017). In China, a study conducted during COVID-19 outbreak found no significant association between generalized anxiety disorder and having more than four chronic diseases (reference group: 2 to 4 chronic conditions) (Wong et al., 2020). In Canada, individuals with obesity and physical multimorbidity were more likely to report anxiety disorders (OR = 2.46 [95% CI 1.46 to 4.16]) (Romain et al., 2019).

### Anxiety and/or depression

Two studies looked at the association of anxiety and/or depression with multimorbidity. In Pakistan (Farooq et al., 2019), found that the presence of multimorbidity (hypertension, obesity, dyslipidaemia, diabetes, heart diseases, stroke, migraines, asthma and chronic obstructive pulmonary disease [COPD], anemia, thyroid disease, diseases of bones and joints, dyspepsia/peptic ulcer, hepatitis B or C, chronic kidney diseases including stones, cancer, and/or disability increased 33% the odds of having anxiety and depressive symptoms (adjusted OR 1.33 [95% CI: 1.11 to 1.58]). Additionally, adults with more than five chronic diseases had increased odds of these symptoms (adjusted OR 2.62 [95% CI: 1.66 to 4.13]) when compared with those with one chronic disease (adjusted OR 0.98 [95% CI: 0.69 to 1.14]) (Farooq et al., 2019). In the USA, DiNapoli et al. (2017) evaluated predictors of having depressive and/or anxiety disorder among middle-aged and older veterans. The study also found significant increased odds of depressive and/or anxiety disorder according to the number of chronic diseases (3–5 chronic diseases: OR = 1.70 [95%CI: 1.21 to 2.40]; 10–13 chronic diseases: OR = 6.62 [95%CI: 4.73 to 9.25]) (DiNapoli et al., 2017).

### Psychosis

Four studies addressed the association between psychosis and multimorbidity. In the Basque Country (Spain), Gabilondo et al. (2017) analyzed a data set of 2,255,406 individuals covered by public health insurance. Of these, 7,331 (0.3%) had a diagnosis of schizophrenia. Compared with individuals without diagnosis of schizophrenia, individuals with schizophrenia had 1.64 odds (95% CI: 1.56 to 1.73; *p* < 0.001) of having one chronic physical illness. Similar odds were found for two (OR 1.63 [95% CI: 1.53 to 1.75], *p* < 0.001; three or more (OR 1.12 [95% CI: 1.05 to 1.19], *p* < 0.001). In addition, the authors found that patients with schizophrenia were more likely to have a diagnosis of hepatitis, HIV, diabetes and any pulmonary disease (Gabilondo et al., 2017). In Bahrain, a case control study compared patients with schizophrenia with those with no serious mental illness. Compared with controls, cases were significantly more likely to have physical comorbidities, but the odds ratio did not vary according to the number of diseases (one disease OR 3.7 [95%CI: 2.0 to 6.9]; two diseases OR 3.3 [95% CI: 1.8 to 6.0]; three or more diseases OR 3.2 [95%CI: 1.4 to 7.7]). In addition, cases were more likely to have obesity, type 2 diabetes, hypertension, cardiovascular disease and musculoskeletal disorders (Jahrami et al., 2017). In a multi-country study, Stubbs et al. (2016) evaluated the risk of multimorbidity according to the severity of psychosis. The findings indicated that patients with a diagnosis of psychosis had higher odds of multimorbidity (4.05 [95% CI: 3.25 to 5.04], *p* < 0.001) compared to those with subclinical psychosis (OR 2.20 [95% CI: 2.02 to 2.39], *p* < 0.001) (Stubbs et al., 2016). In a systematic review and meta-analysis, Rodrigues 2021 found that individuals with psychotic disorder had 1.69 increased risk (RR 1.69 [95% CI: 1.37 to 2.08]) to have multimorbidity, compared with individuals without psychotic disorder (Rodrigues et al., 2021).

### Post-traumatic stress disorder (PTSD)

Two studies assessed the association between PTSD and multimorbidity (Jacob et al., 2018; Peltzer, 2018). In a study conducted in the UK, Jacob et al. (2018) found that PTSD had adjusted odds of 2.47 (95% CI: 1.71 to 3.56, *p* < 0.001) to be associated with physical multimorbidity (cancer, diabetes, epilepsy, migraine, cataracts/eyesight problems, ear/hearing problems, stroke, heart attack/angina, high blood pressure, bronchitis/emphysema, asthma, allergies, stomach ulcer or other digestive problems, liver problems, bowel/colon problems, bladder problems/incontinence, arthritis, bone/back/joint/muscle problems, infectious disease, and skin problems). The number of PTSD symptoms was also associated with increasing odds of multimorbidity (OR 1.14 [95%CI = 1.09–1.20], *p* < 0.001). The authors found that anxiety, depression, and eating disorders were mediators in the association between PTSD and physical multimorbidity (Jacob et al., 2018). In South Africa, a study among patients with tuberculosis found that PTSD was significantly associated with multimorbidity. The association did not change substantially according to the number of non-communicable diseases (one non-communicable disease, adjusted OR 1.42 [95% CI: 1.18 to 1.69]; two non-communicable diseases, adjusted OR 1.79 (1.44 to 2.22); three or more non-communicable diseases, adjusted OR 1.77 [95% CI: 1.30 to 2.41]) (Peltzer, 2018).

### Substance use disorders

Four studies (MacLean et al., 2018; Crawford and Thornton, 2019; Han et al., 2019; Petersen et al., 2019) addressed the association between substance use disorders and multimorbidity. One study included alcohol use disorder (AUD), one was on alcohol and tobacco use disorder (TUD), one on AUD and drug use disorder, and one on drug use disorder (e.g., cannabis, cocaine, methamphetamine, heroin, inhalants, hallucinogens, and prescription drug misuse).

In chronic care patients in South Africa, patients with HIV had twice the odds of having AUD (OR = 2.19 [95% CI: 1.38 to 3.48], *p* < 0.001) compared with those with no HIV (Petersen et al., 2019). MacLean 2018 et al. performed a comparative cohort study of veterans who had received a diagnostic of AUD or TUD in 2012 in the USA. Compared to patient with AUD only, veterans with concurrent conditions had significantly higher odds of hepatic disease (OR 1.14 [95% CI: 1.11 to 1.17]), diabetes mellitus (OR 0.60 [95% CI: 0.59 to 0.61]) and renal disease (OR 0.76 [95% CI: 0.73 to 0.79]). Compared to veterans with TUD only, those with AUD and TUD had significantly higher odds of hepatic disease (OR 2.18 [95% CI: 2.13 to 2.2]), diabetes mellitus (OR 0.81 [95% CI: 0.80 to 0.83]) and renal disease (OR 0.71 [95% CI: 0.68 to 0.74]) (MacLean et al., 2018). In the USA, Han et al. (2019) analyzed the data of a national survey on drug use. Past-year substance use was reported by 16% of adults with one chronic disease and by 13% of adults with two or more chronic diseases. The correlates of past-year substance use among adults with chronic conditions showed an adjusted odds ratio of 1.16 (95% CI: 0.97, 1.39, *p* = 0.11) among adults with 3–4 chronic diseases and 0.67 (95% CI: 0.39, 1.15, *p* = 0.15) among adults with more than five conditions. Results showed lower substance use in individuals with multimorbidity (Han et al., 2019). Another study in the USA among individuals living with HIV found that current alcohol users had a significantly increased risk of multimorbidity compared to never drinkers (adjusted IRR: 1.70 [95% CI: 1.35 to 2.14], *p* < 0.05). The model was adjusted for age, drug use, smoking status, insurance status, HIV duration, cd4 + cell counts, and log viral load. The unadjusted model showed that drug use was also associated with multimorbidity (IRR: 1.22 [95% CI: 1.01 to 1.47], *p* < 0.05) (Crawford and Thornton, 2019).

### Quality assessment

We assessed the quality of three cohorts studies and one case control study. One cohort study obtained fair quality and the remainder were evaluated as poor quality. All cohort studies limited the study population to a selected group of users. The common bias of poor-quality studies were no statement on the comparability of cohorts and adequacy of follow-up. The case-control study was classified as fair quality.

Regarding the cross-sectional studies (20), exposure was assessed through structured interview (55%) or secure record (30%). Outcome was assessed through self-report (65%) or record linkage (35%). The main limitations listed by the authors were cross-sectional study design, self-report data, number, severity and duration of comorbidities included, and other risk factors not included. Details can be found in Table 1.

## Discussion

This systematic review included 26 articles that were published since 2015, the year major publications on the definition of multimorbidity first appeared. Our aim was to frame the relationship between mental disorders and chronic diseases within the multimorbidity framework, in other words, a non-hierarchical framework with multidirectional associations. Such complex structure is harder to fully detect in research and what we found is that we are in a transitional phase where studies are still being designed in a one-to-one type of associative inference, and the studies that ultimately evaluated patterns/groups/clusters reported so distinct groupings that resulted in a very heterogeneous research landscape.

The definition of multimorbidity is still not agreed among researchers. Most studies use a definition based on the number of disorders (2 or more) but some studies use different values (4 or 5 disorders). Even when authors use the same number, there is a problem concerning what diseases are studied. Most studies use a list of conditions, which varies in length and content. Some studies only include chronic conditions, others include acute disorders. The severity of the condition is also not always included. It should be noted, however, that the heterogeneity in our findings can be explained by the broad variety of study types included.

From the small number of studies including mental disorders, the majority (42%) focused on only one disorder (depression). Half the studies only included older individuals (above 50 years), leaving out the young subjects, which are of concern due to high incidence of mental disorders (Ralph et al., 2013). This is surprising given this is a known and important issue acknowledged by the Academy of Medical Sciences in *2015* and the Lancet Psychiatry Commission Blueprint in 2019 (Firth et al., 2019).

The results of this review suggest a strong relationship between depression and multimorbidity. Individuals with multimorbidity had higher odds of having depression or depressive symptoms, with OR ranging between 1.379 (Li et al., 2014) and 1.62 (Arokiasamy et al., 2015). Also, in a systematic review and meta-analysis on multimorbidity and depression, Read et al. (2017) found a risk two times higher of depressive episode in multimorbid patients. The inverse relation was also found, with a multi-country study finding that having depression (subsyndromal or episodic) influenced the odds of having multimorbidity (Stubbs et al., 2017). Although most of these studies were concentrated in samples of older (more than 60 years of age) individuals, they included samples from diverse countries with different income ranges and ethnic groups (Table 1). There is a disproportion of studies concentrated in depression compared to other mental health conditions. Although depression is a highly prevalent disorder (Smith, 2014), there is high comorbidity with other mental health disorders with probable consequences on prevalence of chronic conditions (Steel et al., 2014).

Studies in anxiety disorders found inconsistent results. Whereas, two studies found association between number of chronic conditions and anxiety, with OR that ranged between 1.79 (Gould et al., 2016) and 9.66 (Vancampfort et al., 2017) for more than 5 chronic diseases, another study from China, during the COVID pandemic, found no significant relation between generalized anxiety disorder and more than four chronic diseases (Wong et al., 2020). Two studies focused on anxiety and depression, and both found higher odds of anxiety and depressive symptoms with more chronic diseases (33% in Pakistan and OR of 1.7 in the USA for 3 to 5 chronic conditions) (DiNapoli et al., 2017; Farooq et al., 2019). These studies included younger adults and older individuals.

Individuals with schizophrenia have higher odds of presenting a chronic disease (Gabilondo et al., 2017; Jahrami et al., 2017). Having psychosis increased the chances of having multimorbidity compared to subclinical psychosis (Stubbs et al., 2016). Rodrigues et al. (2021) meta-analysis found a 1.69 risk of multimorbidity among people with psychosis. Studies accounted for different groups of chronic conditions, but diabetes was present in two of them (Gabilondo et al., 2017; Jahrami et al., 2017). These findings are aligned with the large literature showing higher mortality in those with schizophrenia (Walker et al., 2015; Ko et al., 2018; Richmond-Rakerd et al., 2021), which was also observed during the COVID pandemic (Karaoulanis and Christodoulou, 2021).

Both studies with PTSD showed high OR for multimorbidity (Jacob et al., 2018; Peltzer, 2018). The number of PTSD symptoms was related to higher odds of having multimorbidity (Jacob et al., 2018). PTSD was the mental disorder that had fewer studies in this review, its lifetime prevalence in upper-middle income and lower-middle income countries is 2.3 and 2.1 percent, respectively, according to the WHO (Koenen et al., 2017). A previous study reported 17% frequency of comorbidity between PTSD and other coexisting psychiatric disorders, with worse occupational problems, disability and poorer social support among these patients (Solomon and Davidson, 1996). For this reason, the small number of studies evaluating this condition was striking.

Substance use was the subject of four studies. Patients living with HIV had two times the odds of alcohol use disorder (Peterson et al., 2020). In the USA, a study comparing patients with alcohol use disorder and tobacco use disorder showed that the use of both was related with higher odds of having diabetes, renal disease and hepatic disease (MacLean et al., 2018). Koenen et al. (2017) reported that 13% of adults with 2 or more chronic diseases had reported substance use in the last year (Han et al., 2019). Their study found lower rates of substance use among individuals with multimorbidity. This might happen because chronic diseases are more common among older people who are less likely to use substances, and because these individuals stop using substances when they realize they are sick. People in the US living with HIV and current use of alcohol had higher risk of multimorbidity compared to non-drinkers (Crawford and Thornton, 2019). There are not enough studies about the association of substance use and MM. This is particularly important in MM in younger individuals. There is evidence that middle age (40–59) adults with multimorbidity are 1.71 times more likely to be current smokers than non-smokers (Taylor et al., 2010). Tobacco use is related to increased risk for depression, suggesting possible synergistic relation.

This paper should be interpreted in context with its limitations. Comparisons between studies might be hindered due to differences (1) in statistical methods used, (2) in monitored conditions included in each paper, (3) in measuring the conditions, (4) in study type, and (5) due to the broad target population used in the review. Heterogeneity can be in part due to these reasons. This review, as all systematic reviews, only accessed articles published within a specific time frame defined by the authors. Our work is the first to focus on a stricter definition of MM. Previous reviews included primary studies that used MM as a synonym to co-morbidity. Those studies were designed with a notion of hierarchy between disorders that is not compatible with the current MM definition (MacMahon, 2018).

Medicine is organized in specialities that often do not interact seamlessly. Multimorbidity will present a challenge to health service organizations, as it will require better integration of primary care. Furthermore, it will also require changes in how studies are performed. The present review revealed that one approach researchers are taking is to use clustering methodologies. These methods are identifying clinically significant groups, and the non-supervised clustering methods among these have the advantage of becoming starting points to medical automation tools, because of their predictive nature. These algorithms are largely dependent on what conditions are included in the analysis, therefore some kind of standardization will be required in the future, so we can achieve more comparable results. Because of this, we think this field would benefit from the creation of an instrumental scale of diseases/disorders that accounts for number, severity, and weights for clusters that offer higher risk of morbidity/death in multimorbid individuals.

## Data availability statement

The original contributions presented in the study are included in the article/Supplementary material, further inquiries can be directed to the corresponding author/s.

## Author contributions

Material preparation, data collection, and analysis were performed by LC-d-A, FC, NTSF, and ER. The first draft of the manuscript was written by JA, LC-d-A, FC, NTSF, ER, and DM. Contributed reviewing and editing the final version by GL, SD, and MB. All authors contributed to the study conception and design, commented on previous versions of the manuscript, read, and approved the final manuscript.

## Funding

LC-d-A, ER, MB, and DM were funded by the Medical Research Council - UK, Grant No. MR/T03355X/1 during the study development and by the National Institute of Health no. R01MH128911-01 in the later stages of work.

## Conflict of interest

The authors declare that the research was conducted in the absence of any commercial or financial relationships that could be construed as a potential conflict of interest.

## Publisher's note

All claims expressed in this article are solely those of the authors and do not necessarily represent those of their affiliated organizations, or those of the publisher, the editors and the reviewers. Any product that may be evaluated in this article, or claim that may be made by its manufacturer, is not guaranteed or endorsed by the publisher.
